# The Exposome: Embracing the Complexity for Discovery in Environmental Health

**DOI:** 10.1289/EHP412

**Published:** 2016-08-01

**Authors:** Yuxia Cui, David M. Balshaw, Richard K. Kwok, Claudia L. Thompson, Gwen W. Collman, Linda S. Birnbaum

**Affiliations:** 1 Exposure, Response, and Technology Branch, Division of Extramural Research and Training, National Institute of Environmental Health Sciences (NIEHS), National Institutes of Health (NIH), U.S. Department of Health and Human Services (DHHS), Research Triangle Park, North Carolina, USA; 2 Epidemiology Branch, Division of Intramural Research, NIEHS, NIH, DHHS, Research Triangle Park, North Carolina, USA; 3 Population Health Branch, Division of Extramural Research and Training, NIEHS, NIH, DHHS, Research Triangle Park, North Carolina, USA; 4 Division of Extramural Research and Training, NIEHS, NIH, DHHS, Research Triangle Park, North Carolina, USA; 5 Office of the Director, NIEHS, NIH, DHHS, Research Triangle Park, North Carolina, USA

## Abstract

Environmental exposures are ubiquitous and play a fundamental role in the development of complex human diseases. The exposome, which is defined as the totality of environmental exposures over the life course, allows for systematic evaluation of the relationship between exposures and associated biological consequences, and represents a powerful approach for discovery in environmental health research. However, implementing the exposome concept is challenged by the ability to accurately assess multiple exposures and the ability to integrate information across the exposure–disease continuum. On 14–15 January 2015, the National Institute of Environmental Health Sciences (NIEHS) held the Exposome Workshop where a group of international and U.S. scientists from different disciplines gathered to review the state of the science in research areas related to the exposome and to provide recommendations for incorporating the exposome concept into each research area. To move the field forward, the NIEHS is establishing a Children’s Health Exposure Analysis Resource (CHEAR) to provide infrastructure support for access to laboratory and statistical analyses to children’s health studies. It is recognized that incorporating the exposome concept into exposure and environmental health research will be a long journey and will require significant collaborative efforts from different scientific disciplines, nations, and stakeholders.

## Introduction

It is well recognized that both genetic and environmental factors contribute to complex human diseases, and environmental contributions play a major role in disease burden (Schwartz and Collins 2007; Willett 2002). Since 2005, more than 2,300 genome-wide association studies (GWAS) have been published providing valuable insight into the genetic influences on human health and disease (Burdett et al. 2016). However, for many complex human diseases such as cancer, cardiovascular diseases, respiratory diseases, and type 2 diabetes, which are among the leading causes of morbidity and mortality in human populations, genetic variation only explains a modest portion of the disease risk, and much of the disease burden is likely to be attributed to differences in the environment and the interplay between an individual’s genes and the environment (Alwan 2011; Lim et al. 2012; Manolio et al. 2009). One example is that the disease risk for RF (rheumatoid factor)-responsive rheumatoid arthritis associated with the SE gene was significantly increased in the presence of a single environmental factor—tobacco smoking—in populations at risk (Padyukov et al. 2004). Environmental exposures are ubiquitous and arise from a variety of sources including external chemical, physical, biological, and lifestyle factors and endogenous processes influenced by these external stressors such as metabolism, endocrine signaling, and the microbiota (Committee on Human and Environmental Exposure Science in the 21st Century 2012). Traditionally, the effects of environmental exposures have been studied using a targeted, hypothesis-driven approach to investigate the associations between a specific environmental factor and a specific biological end point.

Over the past few decades, studies in exposure science, epidemiology, toxicology, and other related areas, have generated a tremendous amount of knowledge on the impacts of individual, or closely related categories of, environmental chemicals such as arsenic, benzene, dioxins, and many others (Galbraith et al. 2010; Kapaj et al. 2006; White and Birnbaum 2009). However, our understanding of the overall environmental influences on human health and disease is hampered by the fragmentation and compartmentalization of traditional approaches, where only a few environmental factors were selected for study based on an incomplete view of an individual’s total exposure and an imperfect understanding of the temporal and spatial relationship through which these exposures lead to disease. Humans are exposed to numerous potentially harmful environmental chemicals. For instance, about 85,000 chemical substances are currently registered in the United States under the Toxic Substances Control Act (https://www.epa.gov/laws-regulations/summary-toxic-substances-control-act), and an estimated 30,000 of these chemicals are in wide commercial use, but few of these have data on potential health effects or exposure (U.S. EPA 2016; Muir and Howard 2006). As a result of this incomplete view of the exposure–disease relationship, effective disease prevention has been compromised.

**Figure 1:**
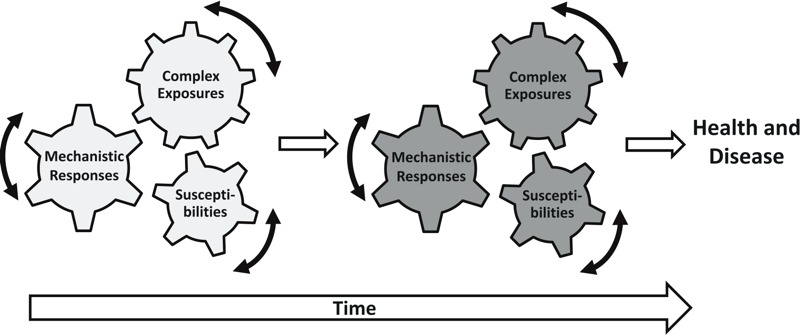
A person’s health and disease status is determined by the combined effects of complex environmental exposures, associated biological responses, and individual susceptibility over time. While individual genomic composition is stable, environmental exposures vary over the time course of an individual’s life as will the way they respond to those exposures or how those responses ultimately manifest themselves as health effects. Adopting the exposome approach is to collect relevant information across all variables, anchor it in meaningful biological consequences and use this for systems analysis and data-driven discovery.

In 2005, Dr. Christopher Wild, Director of the International Agency for Research on Cancer, first defined the exposome as a life course of environmental exposures, including lifestyle factors, that occur throughout a person’s lifespan from the prenatal period onwards. This definition allows for the consideration of multiple environmental contributions to health simultaneously (Wild 2005). To search for the associations between complex exposures and human diseases, Patel et al. (2010) proposed to examine environmental factors that associate with health outcomes in an untargeted, hypothesis-free, manner. Their Environment-Wide Association Study (EWAS) demonstrated the promise of data-driven discovery of unexpected exposure–disease associations and the generation of new hypotheses. Through a systematic evaluation of 266 unique environmental factors that were associated with the clinical status of type 2 diabetes, they were able to identify significant associations for several environmental factors, such as the pesticide metabolite heptachlor epoxide, vitamin γ-tocopherol, and polychlorinated biphenyls (PCBs); however, further investigation is needed to ascertain causality. In contrast to traditional approaches, the exposome approach changes the perspective of research away from one focused on specific exposure–disease relationships to one that is agnostic and uses expanded data collection over a broader range of environmental variables across time and space, as well as data integration across the entire exposure–disease continuum—from external exposure and internal dose to associated biological perturbations. A growing body of evidence suggests that various biological and social–environmental factors throughout the life course, especially during early development, can interactively and cumulatively influence health and disease in people by inducing physiological and epigenetic changes that have long-term consequences. This has been well described in the concept of developmental origins of health and disease (DOHaD) and the life-course epidemiology approach (Gluckman et al. 2007; Lynch and Smith 2005). To unravel the underlying mechanisms of complex human diseases, it is critical to understand both a person’s environmental exposure history, which is highly variable and dynamic through a lifetime, and the timing of those exposures (Figure 1). Systems approaches are required to integrate these diverse types of information. The systematic evaluation of the relationship between exposure and associated biological consequences provides valuable insight into windows of susceptibility in disease development, as well as how environmental and genetic factors interact with each other to contribute to human health and disease.

## Discussion

Taking full account of the exposome and incorporating it into research is challenging in many aspects, including the ability to accurately assess multiple exposures and the ability to integrate information. Currently, large exposome initiatives have incorporated the complexity of the exposome into certain focal areas such as prenatal or early childhood exposure (Buck Louis et al. 2013; Vrijheid et al. 2014). The key to implementing the exposome concept in environmental health research is to embrace the complexity of environmental exposures by recognizing that these exposures are not limited to synthetic chemicals but also include nutrients, drugs, products of infectious agents, microbiome metabolites, physical stress, and psychosocial stress and that exposure and response may vary at multiple levels and scales. Oversimplifying environmental exposures could lead to misclassification and confounding, which are some limitations in current environmental epidemiology studies.

### Technological Challenges in Implementing the Exposome Concept

One major demand of incorporating the exposome concept into environmental health research is advancing our technological capabilities and infrastructure. Although many tools and technologies for investigating the exposome exist, researchers must overcome many practical barriers to apply them in large-scale population. Recent advances in the development of omics technologies such as transcriptomics, proteomics, metabolomics, and epigenomics, allow for high-content data to be readily collected from various biological samples of interest, and they offer powerful tools for a global quantitative analysis of exposure and response. However, applying these technologies on a large scale requires careful study design, standardized protocols, and appropriate interpretation of results to overcome such issues as reproducibility and intra-individual variation. Novel data integration tools are also needed to integrate such heterogeneous and extensive omics data. The emergence of wearable sensors and smart phone technologies provides possibilities for real-time monitoring of external environmental exposure at the personal level (Balshaw and Kwok 2012). Yet, obstacles preventing the implementation of these technologies in routine use still remain. In particular, versatile devices with the capability of measuring multiple analytes are still lacking. As a result, applying these devices in large-scale studies is challenged by the financial cost and burden to both investigators and participants. Another significant challenge in implementing the exposome concept is the management and analysis of the extensive data output that would be produced from the measurement of both exposure and response (i.e., big data). Given the scope and complexity of the exposome, as well as the high-throughput and high-content technologies that will be employed, exposure and health data are anticipated to accumulate rapidly, and data sharing will become an important component. As data sharing becomes more common and accepted, there will be significant challenges including financial costs and ethical issues in addition to technical difficulties such as the use of different data standards and platforms. Analyzing data is an even greater challenge considering the high dimensionality (number of variables and repeated measurements over time) and the diverse types (external and internal measurements, molecular and phenotypic responses, temporal and spatial variables) of the data, as well as the different statistical and informatics needed to integrate across all the information.

The National Institute of Environmental Health Sciences (NIEHS) recognizes the complexity of environmental exposures and the potential of the exposome concept as well as the technological barriers to implementing it. As such, our efforts over the past decade have emphasized the development of the technology, capacity, and infrastructure to assess environmental exposures and their contributions to human health. Even before the initial development of the exposome concept, the NIEHS invested in the advancement of metabolomics technologies in the study of metabolic and regulatory pathways that are perturbed by environmental exposures, which can lead to the development of toxicity and disease (NIEHS 2004). In 2006, the NIEHS established the Exposure Biology Program (EBP), as part of the National Institutes of Health (NIH) Genes and Environment Initiative, to focus on developing wearable and field-deployable sensor systems to enable more precise, accurate, and quantitative assessments of environmental exposure to chemicals, dietary intake, physical activity, psychosocial stress, or substance abuse at the personal level with temporal and spatial resolution. The EBP also included focused efforts to link complex environmental exposures with pathway level biological response indicators to develop more informative biomarkers linking exposure and response. The ultimate goal is to develop versatile systems with multi-component detecting capacity that could be applied in epidemiological research settings to integrate exposures and stressors across these areas and to understand the development and progression of complex disease in the context of the individual’s exposure and response. The NIEHS continues to support both technology development and the validation and demonstration of novel devices in environmental health research. It is also a key leader in the NIH Common Fund metabolomics program, which was initiated in 2012 to support the development of next generation technologies and provide infrastructure support as well as training to the application of metabolomics in human health research. In 2013, NIEHS funded its first effort explicitly linked to the exposome—a P30 Environmental Health Sciences Core Center at Emory University—with a focus on providing a more comprehensive assessment of environmental exposures by utilizing exposome-based concepts and approaches (HERCULES 2016). To understand the complex interplay between genes and environmental factors in complex human diseases, the NIEHS also led research efforts in the development of novel statistical and bioinformatics approaches for identifying gene–environment (G × E) interactions and the application of these methods in population studies.

### The NIEHS Workshop on Exposome

The NIEHS organized a workshop that was held on 14–15 January 2015 to raise trans-disciplinary awareness of the exposome concept and to promote broader incorporation of the concept in the study of environmental health. This workshop allowed scientists from the critical disciplines needed to implement the exposome concept to interact and share information about the state of the science. The different areas of scientific expertise represented at this workshop included clinical medicine, epidemiology, toxicology, exposure biology, pharmacology, and bioinformatics. Five work groups were formed to review the state of the science in different areas related to the exposome: *a*) external exposure assessment (assessing the exposome external from the organism), *b*) biomonitoring (assessing the exposome using biological samples) (Dennis et al. 2016a), *c*) biological response and impact (assessing biological consequences related to exposures) (Dennis et al. 2016b), *d*) epidemiology (how to incorporate the exposome concept into population studies), and *e*) data science (data and analytic challenges in exposome-based studies). These groups were tasked with assessing the state of the science in their domain and to formulate an initial series of recommendations with the overarching goal of identifying opportunities and common challenges in implementing the exposome concept and advancing environmental health research. More detailed and area-specific discussions and recommendations will be provided in a series of articles that will be published in the near future. Three major crosscutting areas were identified as top priorities: infrastructure support, technology advancement, and strategies to promote the exposome concept.

The comprehensive nature of the exposome necessitates that implementing exposome research will be an inherently interdisciplinary activity that will also require the engagement of the international research community. There is consensus among scientists across different disciplines that there is a critical infrastructure need for an exposome clearinghouse to support comprehensive exposure assessment on a large scale, promote data sharing and interdisciplinary collaboration, and to identify opportunities and common challenges in implementing the exposome concept. A centralized infrastructure can provide both the range of technical expertise needed and a driver for more broadly disseminated scientific expertise by serving as an openly accessible resource of tools, methods, and data for the environmental health science community. It can also serve as a test bed for evolving technologies by providing opportunities for refining and validating the new technologies before they are disseminated.

Expanding current technological capabilities is another crosscutting area requiring significant efforts. While whole genome sequencing and GWAS have enabled large scale, untargeted discovery of genetic contributions to disease susceptibility, characterization of the exposome is a much greater challenge because of its broader scope and highly dynamic nature. Our ability to characterize environmental exposure, especially during critical windows of susceptibility, is far behind our ability to characterize genomic variation and constrains the large-scale incorporation of environmental factors into population studies. An array of complementary approaches and technologies are required for the assessment of exposures both in biological samples and in a person’s environment, such as exposure modeling, sensors, questionnaires, biomonitoring, and omics technologies. Developing novel tools and methods for detection and quantitation of environmental stressors, as well as refining, validating, and scaling up existing tools, are all crucial to enable incorporation of the exposome concept into environmental health research. Many tools currently exist and can be applied through a centralized infrastructure such as biomonitoring of various internal exposures through targeted analyses; others are under development and require extensive validation and refinement in order to be successfully implemented such as real-time monitoring of external environmental exposures through wearable sensors or smart phone-based technologies. An iterative process is needed to assure that these tools are applied and used to drive further development of subsequent generations of more powerful, usable, and cost-effective tools. Exploration of innovative untargeted analyses and alternative exposure assessment platforms is also key to advancing our understanding of the exposome. This untargeted discovery requires novel computational methods, big data mining and bioinformatics tools for analyzing the heterogeneous and multi-dimensional and exposure data as well as the integration with genomic and health outcome data.

In practice, identifying case studies or research areas that can be greatly advanced by incorporating the exposome concept will help promote broader application of the concept. This can be achieved by encouraging secondary analyses of banked samples from existing cohort studies, especially unique national and international cohorts with high or unusual exposures, mining of existing datasets from an exposome point of view, establishing policies to promote data sharing, educational and outreach opportunities, as well as training a new generation of environmental scientists.

### A Pathway Forward: The NIEHS CHEAR Program

Building on previous success in the EBP, gene–environment (G × E) interactions and various efforts in expanding exposure assessment capacity, the NIEHS is establishing a Children’s Health Exposure Analysis Resource (CHEAR) to provide infrastructure support for access to comprehensive environmental exposure analysis of biological samples related to children’s health research (NIEHS 2016). Children are among the most vulnerable populations to the negative health outcomes associated with a variety of environmental exposures due to their rapid development and often higher-exposure burdens due to their smaller size and behaviors as well as underdeveloped detoxification systems. Environmental exposures during critical early developmental life stages such as *in utero* or neonatal periods can have long-lasting effects that can influence health and disease later in life. Researchers must incorporate a broad array of environmental exposures into their studies to fully understand the complexity of children’s health (i.e., the combined effects of genetic and environmental factors and gene environment interactions). CHEAR contains three components: a network of laboratories, a data and analytics support resource, and a coordinating center. CHEAR will leverage both existing and evolving technologies in biomonitoring, including both targeted and untargeted approaches, to provide comprehensive and state-of-the-art analyses of a wide array of environmental factors and biological response indicators. CHEAR intends not only to empower children’s health research by integrating exposure data into existing genomic and clinical data but also to expand and improve current analytical capabilities to explore the full breadth of the exposome. The CHEAR data center will also lead a significant effort in developing community-based data standards and support the development of ontologies and metadata standards to promote broader data sharing and integration in the larger environmental health research community.

An NIH companion program, the Pediatric Research Using Integrated Sensor Monitoring Systems (PRISMS), which is led by the National Institute for Biomedical Imaging and Bioengineering, has also been announced (NIBIB 2016). This program aims to establish capabilities for characterizing the external environment in pediatric studies. Together, CHEAR and PRISMS will provide complementary technologies to enable the characterization of the full breadth of the exposome in children’s health research and allows systematic analyses and data-driven discovery of the dynamic relationships between genes, environment, and human health as well as how such relationships can be modified by human behavior and personal activities over time. NIEHS is committed to proactively engaging the scientific community and stakeholders to develop capacities to incorporate the exposome concept into human health research, creating opportunities to foster collaborations, and advancing our understanding of the environmental contributions to adverse human health outcomes in order to reduce and prevent disease and disability.

## Conclusions

A decade after the original publication of the exposome concept, followed by years of fruitful discussions on the challenges of implementing it in research, the time is ripe to use the concept to guide study design, to understand the complex nature of exposure, and to embrace that complexity when exploring the relationship between exposure and human health and its underlying mechanisms. It is vital to identify case studies where we can refine and improve epidemiological findings via incorporation of the exposome concept, including expanded exposure measurements, increased understanding of exposure variations across time and space, integration of external measures, biomonitoring and biological response, and comprehensive analysis of the gene environment interplay. One example is the Longitudinal Investigation of Fertility and the Environment (LIFE) study, which examines the relationship between persistent environmental pollutants and fecundity. This study is a prospective cohort of couples who completed daily journals on lifestyle factors and provided biospecimens for assessment of exposure to more than 60 persistent environmental chemicals including organochlorine pesticides, polybrominated diphenyl ethers, PCBs, and perfluorochemicals (PFCs) (Buck Louis et al. 2013). The incorporation of exposure metrics and biospecimens across time makes it a valuable resource to explore the exposome. It is also important to recognize that the exposome approach comprises multiple stages including a discovery stage for hypothesis generation and follow-up studies for further validation and disease prevention. In this sense, the exposome approach is not a replacement of traditional approaches. Rather, it is complementary and produces synergy by unifying the strengths of different scientific domains.

In order to promote an even broader and more sustainable incorporation of the exposome concept into research, it is critical to partner with global and international organizations that include industry and the private sector so that the exposome concept can be integrated into their program development. By doing so, we can enhance research in the exposome paradigm—more precisely, we can pinpoint exposures that contribute to disease and disability—and continue to advance our understanding of environmental health to promote healthier lives for all.
